# Association of Polymorphisms of *MASP1/3*, *COLEC10*, and *COLEC11* Genes with 3MC Syndrome

**DOI:** 10.3390/ijms21155483

**Published:** 2020-07-31

**Authors:** Gabriela Gajek, Anna S. Świerzko, Maciej Cedzyński

**Affiliations:** Laboratory of Immunobiology of Infections, Institute of Medical Biology, Polish Academy of Sciences, 93-232 Lodz, Poland; aswierzko@cbm.pan.pl (A.S.Ś.); mcedzynski@cbm.pan.pl (M.C.)

**Keywords:** 3MC syndrome, *MASP1/3*, *COLEC11*, *COLEC10*, MASP-1, MASP-3, CL-K1, CL-L1

## Abstract

The Malpuech, Michels, Mingarelli, Carnevale (3MC) syndrome is a rare, autosomal recessive genetic- disorder associated with mutations in the *MASP1/3*, *COLEC1,1* or *COLEC10* genes. The number of 3MC patients with known mutations in these three genes reported so far remains very small. To date, 16 mutations in *MASP-1/3*, 12 mutations in *COLEC11* and three in *COLEC10* associated with 3MC syndrome have been identified. Their products play an essential role as factors involved in the activation of complement via the lectin or alternative (MASP-3) pathways. Recent data indicate that mannose-binding lectin-associated serine protease-1 (MASP-1), MASP-3, collectin kidney-1 (collectin-11) (CL-K1), and collectin liver-1 (collectin-10) (CL-L1) also participate in the correct migration of neural crest cells (NCC) during embryogenesis. This is supported by relationships between *MASP1/3*, *COLEC10,* and *COLEC11* gene mutations and the incidence of 3MC syndrome, associated with craniofacial abnormalities such as radioulnar synostosis high-arched eyebrows, cleft lip/palate, hearing loss, and ptosis.

## 1. Introduction

The Malpuech, Michels, Mingarelli and Carnevale syndrome [1,2,3,4] is commonly called 3MC syndrome. In 1989, Carnevale et al. reported a phenotype consisting of downslanting palpebral fissures, ptosis of the eyelids, periumbilical depression, hypertelorism, radioulnar synostosis, and developmental delay in two Italian siblings (MIM 265050). In 1996, two sisters with similar ocular, facial, skeletal, and abdominal defects, but with normal intelligence, were reported by Mingarelli et al. (also MIM 265050). Since the clinical picture of patients suffering from Carnevale and Mingarelli syndromes overlapped with Michels (MIM 257920) and Malpuech syndromes (MIM 248340), it was suggested that all four disorders should be reclassified into one “3MC syndrome”. It is a rare, autosomal recessive genetic disorder, characterized by a wide spectrum of developmental abnormalities that could include high-arched eyebrows, cleft lip/palate, hearing loss, short stature, umbilical hernias/omphalocele, and urogenital abnormalities. The prevalence of 3MC syndrome is unknown. The largest number of affected persons is located in the Middle East. 3MC syndrome disorders are caused by mutations in the mannose-binding lectin-associated serine protease (*MASP)1/3* [5], *COLEC11* [6], or *COLEC10* [7] genes. The number of 3MC patients carrying known mutations in these three genes reported so far still remains rather small but the disease is more likely to occur in families with consanguineous parents. In total, forty-six 3MC patients from 34 families with mutations in the above-mentioned genes have been diagnosed. Among them, 26 persons from 20 families had mutations in the *MASP1/3* (Table 1), 17 individuals from 12 families had mutations in the *COLEC11* (Table 2), and three patients from two families had mutations in the *COLEC10* gene (Table 3). Those mutations abort or impair function of their corresponding proteins, resulting in defective control of cell migration at an early stage of embryonal development [6]. Impaired cell migration interferes with ontogenesis of tissues and organs and leads to the various abnormalities manifested. The proteins expressed are also factors involved in the activation of complement via the lectin pathway, an important branch of the innate immune system [6,8]. It was suggested that dysfunction of the lectin pathway can be compensated by other defense mechanisms, which seems to explain why immune system dysfunctions are not part of the 3MC syndrome. 

## 2. The *MASP1/3* Gene and Its Products—MASP-1, MASP-3, and MAp44

### 2.1. Gene and Protein Structure

The MASP1/3 gene is located on chromosome 3 (3q27-28). It contains 76 kbp and encompasses 18 exons. This gene encodes for two enzymes, MASP-1 and MASP-3 (mannose-binding lectin-associated serine proteases) as well as the non-enzymatic MAp44 (mannose-binding lectin-associated protein, 44 kDa) [9]. All three MASP1/3 alternative splicing products share four N-terminal domains, encoded by exons 2–8: CUB1-EGF-CUB2-CCP1 (explained below), while exon 1 encodes the 5′-untranslated region [8]. Exons 10 and 11 encode the CCP2 domain, common to MASP-1 and MASP-3. Single exon 12 is responsible for the serine protease (SP) domain of MASP-3, whereas exons 13—18 are responsible for the corresponding MASP-1 fragment. The ninth exon encodes the unique C-terminal 17 AA sequence of MAp44. According to the NCBI database a fourth splice variant has been recorded, however the resulting RNA does not constitute mRNA, and presumably is degraded through the nonsense-mediated mRNA decay pathway [10].

**Figure 1:**
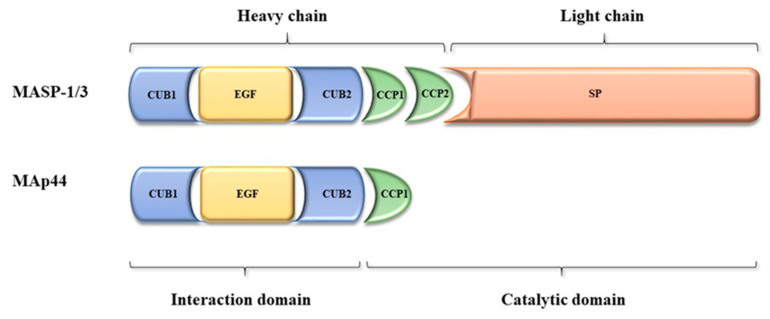
Scheme of mannose-binding lectin-associated serine protease-1 (MASP-1), MASP-3, and mannose-binding lectin-associated protein, 44 kDa (MAp44) protein structures (acc. to Gaboriaud et al., 2013 [16].

Therefore, MASP-1 and MASP-3 are proteases composed of six well-characterized domains, of which five constitute the heavy chain while the sixth (SP) constitutes the light chain. Upon activation of proenzymes, the peptide bond between them is cleaved and both chains remain connected via a disulphide bond [11,12]. The CUB (C1r/C1s-Uegf (uchrin epidermal growth factor)-BMP (bone morphogenic protein)) and EGF (epidermal growth factor) domains are responsible for forming MASP/MAp44 complexes with such pattern-recognition molecules (PRM) as collectins and ficolins [13]. It should be stressed that most of the proteins possessing the CUB domain are involved in developmental processes, such as bone morphogenetic protein 1, the dorso-ventral patterning protein tolloid, a family of spermadhesins, and the neuronal recognition molecule A5 [8,14]. The CCP (complement control protein) domains are common to a variety of complement factors [15]. The serine protease (SP) domain with catalytic activity is characteristic for chymotrypsin-like proteases [8]. There are several single nucleotide polymorphisms of the MASP1/3 gene strongly associated with protein serum levels. Among them, homozygosity in intron 8 (rs3774275, G/G) was associated with decreased MASP-3 but increased MASP-1 and MAp44. Variant alleles for rs698090 and rs67143992 are associated with an increase in MASP-1 and MAp44 and a decrease in MASP-3, while variant alleles of rs72549154 and rs35089177 result in decreases of MASP-1 and MAp44 and an increase of MASP-3 [9], Figure 1.

### 2.2. Tissue Expression of the MASP-1, MASP-3, and MAp44

MASP-1 is mainly expressed in the liver although specific mRNA was also found in the brain and cervix. Similarly, the major site of MASP-3 synthesis is the liver but its expression was also noted in a variety of other sites, including the colon, bladder, and uterus. MAp44 is synthesized in the heart while weaker expression takes place in the liver, brain, and cervix [10,17,18]. The expression of MASP-3 has been considered ubiquitous compared with other isoforms [10]. These results emphasize the importance of alternative splicing mechanisms in the regulation of expression in different tissues. The widespread expression of the MASP1/3 gene may indicate local functions of its products MASP-1, MASP-3, and MAp44. The median serum levels of MASP-1, MASP-3, and MAp44 in healthy adults were found to equal 10.8, 6.7 and 2.2 µg/mL, respectively. Concentrations of MASP-1 and MASP-3 showed gender differences—the first mentioned was higher in women while the second was higher in men [19]. Moreover, MASP-3 and MAp44 also correlate with age [19].

### 2.3. Biological Activities of the MASP-1, MASP-3, and MAp44

Mannose-binding lectin-associated serine protease-1 (MASP-1) functions as a factor involved in the activation of complement, coagulation, and kallikrein–kinin systems. As mentioned, it exists in the form of a zymogen occurring in complexes with some collectins (MBL, collectin liver-1 (also known as collectin 10, CL-10), collectin kidney-1 (or collectin 11, CL-11)), and ficolins (ficolin-1, -2, -3 or M-, L-, H-ficolin, respectively). MASP-1 is auto-activated when the complex binds to the target, recognized by PRM (collectins and ficolins, as lectins recognize mainly polysaccharides or glycoconjugates expressed on the surface of pathogens or abnormal self-cells). MASP-1 then cleaves MASP-2 zymogen (another protease of the MASP-2 family, encoded by the distinct MASP-2 gene), which next activates complement C4 factor. Both MASP-1 and MASP-2 further activate C2 forming C3 convertase (C4bC2a) (identical to that of the classical pathway) [20]. Moreover, MASP-1 is able to cleave fibrinogen, factor XIII, prothrombin, and thrombin-activatable fibrinolysis inhibitor (TAFI) thus promoting coagulation [21,22,23,24]. The clots can gather around microbes and hinder their spreading [25]. Furthermore, the high-molecular-weight kininogen was reported to be another MASP-1 substrate. Its digestion enables release of bradykinin, a pro-inflammatory mediator of the contact system [26]. Moreover, MASP-1 can activate endothelial cells [27], enhance endothelial E-selectin expression [28], and increase the production of interleukins IL-6 and IL-8 [29].

MASP-3 is believed to contribute to the activation of complement via the alternative pathway through pro-factor D cleavage [20]. Another substrate is insulin-like growth factor-binding protein 5 (IGFBP-5) that modulates the effects of IGF on cell survival, differentiation, and proliferation [30].

MAp44 functions as an inhibitor of the complement pathway by competing with MASP for PRM-binding sites [31]. It moreover contributes to regulation of cardiac development [32].

Some clinical associations of MASP-1, MASP-3, and MAp44 other than 3MC have been reported. For example, Weinschutz Mendes et al. (2020) found that polymorphisms influencing serum concentrations of MASP-3/MAp44 modulate susceptibility to leprosy [33]. Larsen et al. (2019) showed that low MASP-1 concentrations were associated with clotting disorders in patients with septic shock [34]. Michalski et al. (2019) demonstrated that children undergoing surgical correction of congenital heart disease with low pre-operative MAp44 concentration were more likely to suffer from post-operative complications such as systemic inflammatory response syndrome (SIRS), renal failure, multiorgan dysfunction (MODS), or low cardiac output syndrome (LCOS), while high MASP-1 and low MASP-3 were often associated with fatal outcome [35].

## 3. *COLEC10* and *COLEC11* Genes and Their Products, CL-L1 and CL-K1

### 3.1. Genes and Protein Structures

Collectins constitute a group of 400–800 kDa oligomeric, calcium-dependent lectins consisting of basic subunits that are triplets of 30–35 kDa polypeptide chains. Collectin liver-1 (collectin-10) (CL-L1) and collectin kidney-1 (collectin-11) (CL-K1) are closely related, both structurally and functionally [36]. The collectin polypeptide chain consists of an N-terminal region containing cysteine, a collagen-like domain with Gly-Xaa-Yaa repeats (where Xaa and Yaa represent any amino acid residues), a neck region, and a C-terminal carbohydrate-recognition domain (CRD). The CRD is responsible for interactions with sugar residues on microbial surfaces or aberrant host cells, whereas the collagen-like domain forms complexes with proteins of the MASP family as well as interacting with cell receptors. CL-L1 and CL-K1 form heterocomplexes called CL-LK, existing in blood. Each molecule is stabilized by a disulphide bridge (created by Cys residues present in N-terminal domains) with two CL-K1 and one CL-L1 polypeptide chains [37]. CL-K1 is present in species ranging from the zebrafish to humans (with protein identities of full length protein: 72–98%). The protein identity between the CRDs of CL-K1 and CL-L1 reaches 54%. This compares with only 25–32% for the corresponding domains of other collectins. [38].

The human *COLEC10* gene is localized to chromosome 8 (8q23–q24.1) and includes six exons. The first one encodes for the untranslated region, the N-terminal domain, and the first Gly-Xaa-Yaa sequence of the collagen-like region. The rest of the collagen-like domain is encoded by exons 2–5. Exon 5 is also responsible for the sequence of alpha-helical coiled-coil neck region while exon 6 encodes for the CRD domain [39]. Twenty polymorphic sites were identified in the promoter, exons, and flanking regions of the *COLEC10* gene [40]. None of promoter polymorphisms were found to influence CL-L1 serum level. The +3654 C>T polymorphism (rs149331285, exon 5) affects the protein structure due to an amino acid exchange (p.Arg125Trp). Heterozygosity was demonstrated to be associated with significantly-higher CL-L1 serum concentration, compared with C/C homozygosity. Moreover, the -161/-157AAAATdel (rs148350292) may disturb the binding of several transcription factors essential for liver development or immune response modulation [40].

**Figure 2:**
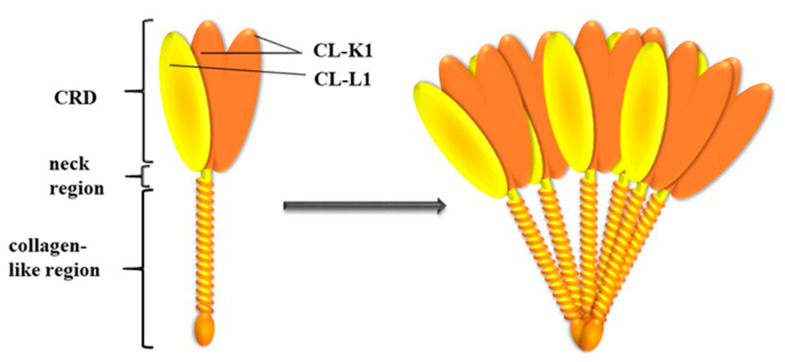
Subunit and oligomeric structure of CL-LK. A total of three polypeptide chains of (two of CL-K1 and one of CL-L1) join to form a heteromeric subunit, which may further oligomerize into structures ranging from dimer to hexamer (acc. to Hansen et al., 2018 [41]).

The *COLEC11* gene is localized to chromosome 2 (2p25.3). It includes eleven exons, of which seven encode for the dominant isoform CL-11-1. With the exception of the first one that encodes the untranslated region, the other six exons are arranged and encode similar regions to that of the *COLEC10* gene [39]. Several variations in the promoter, exons and introns have been identified. Among them, promoter polymorphism -9570 C>T (rs3820897) was shown to influence CL-K1 serum level. The non-synonymous SNP (p.His219Arg) in exon 7 (+39618 C>G, rs7567833) has no impact on protein concentration in serum, however it is suspected to influence ligand binding [40], Figure 2.

### 3.2. Tissue Expression of the Collectin-Liver 1 and Collectin-Kidney 1

The tissue expression of both CL-K1 and CL-L1 is ubiquitous. The highest expression of the first mentioned was found in the liver, kidney, adrenal gland, ovary and gallbladder (and to a lower extent, in the lung, ovary, testis, and retina) whereas expression of CL-L1 was found mainly in the liver and adrenal gland [38,41,42]. In the adrenal gland, CL-K1 was reported to be expressed in cells of all three layers—spinal, banded, and reticular, whereas in the kidney, it is expressed in the distal canals, as well as the glomeruli and proximal tubules [38]. In the ovary, it is associated with granulosa and theca lutein cells while in the testis, the main expression sites are seminiferous tubules [38]. In the liver, the expression of both CL-K1 and CL-L1 is associated with hepatocytes [38,43]. Furthermore, high expression of both collectins was detected in placenta [42]. Average human CL-L1 serum level was estimated to be 0.5 μg/mL, while CL-K1 was estimated to be approximately 0.4 μg/mL. Their concentrations are strongly correlated [19]. No significant differences with sex, age (18–70 years), or diurnal variations were found [19,40,44,45].

### 3.3. Biological Activity of Collectin-Liver 1 and Collectin-Kidney 1

CL-K1 was reported to recognize certain Gram-negative bacteria (*E. coli* O126 and O60, *Pseudomonas aeruginosa,* and *Klebsiella pneumoniae*), mycobacteria (*Mycobacterium tuberculosis*), fungi (*Candida albicans* and *Saccharomyces cerevisiae*), and viruses (influenza A virus) [38,42,46]. Surface structures identified amongst those recognized by CL-K1 include lipopolysaccharides (LPS core region of *E. coli* mutants of Ra and Rd, but not Re chemotypes), *S. cerevisiae* mannan, mycobacterial mannosylated lipoarabinomannan (ManLAM) [42,46], and HIV gp120 [47]. Venkatraman Girija et al., (2015) demonstrated the affinity of CL-K1 to mannose-rich glycans containing the disaccharide Man(α1-2)Man as terminal motif [47].

The microbial structures recognized by CL-L1 are not yet established, but formation of heterodimers with CL-K1 could possibly lead to an increased range of interactions with microorganisms, as well as to their higher affinity. Thus, CL-LK is characterized by high affinity to d-mannose (d-Man), n-acetyl-d-glucosamine (d-GlcNAc), d-galactose (d-Gal), l- and d-fucose (l-Fuc, d-Fuc), and n-acetyl-d-mannosamine (d-ManNAc) [41]. CL-LK, due to cooperation with MASP, may initiate activation of complement via the lectin pathway (and cross-talk with the coagulation cascade). They may also aggregate and opsonize microorganisms contributing to enhanced phagocytosis [48].

Through the collagen-like domain, but also via its CRD, CL-K1 binds to mammalian and bacterial DNA in a calcium-independent manner [49]. It also binds to DNA exposed on apoptotic/necrotic cells and may facilitate their clearance, preventing formation of anti-DNA antibodies. The CL-K1–DNA interaction may lead to complement activation. However, it is much weaker compared to other collectins, probably due to different degrees of protein oligomerization [50].

Only a few reports concerning CL-LK disease associations and the clinical significance of CL-L1/CL-K1 have been published to date. Increased blood CL-L1 levels were observed at early stages of acute liver failure and cirrhosis. Smedbråten et al. (2017) reported that high plasma concentrations of CL-L1 and CL-K1 at the time of transplantation are correlated with increased mortality in kidney transplant recipients [51]. Troldborg et al. (2018) found lower CL-L1 and CL-K1 levels in systemic lupus erythematosus (SLE patients), compared with healthy controls, however no association with SLE disease activity index (SLEDAI) score was found [52]. Storm et al. (2014) reported that CL-L1 may significantly distinguish between patients with colorectal cancer (CRC), patients with adenomas, and individuals without neoplastic bowel lesions [53]. Higher levels of CL-L1 were associated with lower odds of CRC [53]. Świerzko et al. (2018), reported higher serum levels of CL-LK complex in multiple myeloma patients compared with healthy individuals [45]. Farrar et al., (2016) showed that deficiency of CL-K1 is protective in the case of ischemia, preventing tubular damage and loss of renal function [54]. Similar conclusions were also drawn by Wu et al. (2018), who demonstrated that CL-K1 plays a harmful role in the development of tubulointerstitial fibrosis by leukocyte chemotaxis and the impact on the proliferation of renal fibroblasts [55]. Koipallil Gopalakrishnan Nair et al. (2015) observed an approximately 3-fold increase in CL-K1 and CL-L1 transcript expression in human ectopic endometriotic mesenchymal stem cells (MSCs) in comparison with eutopic MSCs [56].

## 4. Associations of *COLEC10*, *COLEC1,1* and *MASP1/3* Gene Mutations with 3MC Syndrome

Association of *MASP1/3* gene polymorphisms with 3MC were first reported by Sirmaci et al. (2010), in three individuals from two related Turkish families. They found a missense c.2059 G>A (p.Gly687Arg) mutation specific to the MASP-3 isoform and nonsense c.870 G>A (p.Trp290*) in exon 6, which is shared by all gene products. In silico results suggested that the p.Gly687Arg mutation may be damaging for the MASP-3 catalytic domain [5]. Other abnormalities of the same gene were identified by Rooryck et al. (2011). They described three homozygous missense mutations, all located within exon 12, encoding the MASP-3 protease domain, in the 3MC-syndrome-affected patients (c.1489C>T p.His497Tyr, c.1888T>C p.Cys630Arg, and c.1997G>A p.Gly666Glu). All of them are predicted to affect protein activity [6]. Atik et al. (2015) reported two splice site mutations, c.1012 - 2 A>G in one proband and c.891 + 1 G >T in two propositi, as well as three missense mutations, c.1451 G>A (p. Gly484Glu), c.1657 G >A (p. Asp553Asn), and c.1987 G >T (p. Asp663Tyr) in three other propositi. Splice site mutations affect both MASP-1 and MASP-3, while missense mutations affect MASP-3 only. A total lack of lectin pathway activity and a 2.5-fold lower alternative pathway activity in a proband homozygous for c.891 + 1 G > T were found [57]. Urquhart et al. (2016) identified three other mutations within the *MASP1/3* gene (c.9 G>A p.Trp3*, c.547 G>T p.Val183Leu, and c.760 A>T p.Leu254*) [58]. The missense alteration, p.Asp663Tyr (reported also, as mentioned, by Atik et al. (2015)) probably changes the enzymatic activity of the catalytic domain [58]. Pihl et al. (2017) described the proposita from a German family with a homozygous missense mutation c.1993G.A (p.G665S) in exon 12 of the *MASP1/3* gene, confirming the clinical diagnosis of 3MC syndrome. The serum concentration of MASP-3 in this patient was lower than the median for a healthy population [59]. Later, Graul-Neumann et al. (2018) reported a Turkish individual carrying a deletion of 2306 bp (c.1895_*1602+411del). It affects the terminal part of exon 12 and accordingly the C-terminal serine protease domain specific to MASP-3 [60]. A separate study of two individuals from a Turkish family was conducted by Basdemirci et al. (2019). They identified a new missense homozygous mutation c.2111T>G (p. Val704Gly) (NM_139125.3) in exon 11 of the relevant gene [61]. Çakmaklı et al. (2019) found the afore-mentioned missense c.1987G>T; (p.Asp663Tyr) *MASP1/3* mutation (in a homozygous state) in a patient from Syria [62]. In summary, so far 16 3MC-syndrome-associated mutations in the *MASP-1/3* gene in 26 individuals have been reported (Table 1). The majority of them (11) affect the serine protease domain of MASP-3.

Rooryck et al. (2011) demonstrated an association of homozygosity for three non-synonymous mutations and one in-frame deletion in the *COLEC11* gene: c.496 T>C (rs387907075, p.Ser169Pro, exon 8), c.45delC (RCV000023960, p.Phe16Serfs X85, exon 2), c.610 G>A (rs387907076, p.Gly204Ser, exon 8), and c.648-650delCTC (RCV000023962, p.Ser217del, exon 8). Moreover, they identified a 27-kb homozygous deletion encompassing exons 1–3 of *COLEC11*, predicting partial loss of the collagen-like domain, and complete loss of the N-terminal (cysteine-rich) domain. Furthermore, a homozygous single-base deletion (c.300delT/G101VfsX113 in exon 6) was suggested to lead to premature termination of the *COLEC11* gene product [6]. Gardner et al. (2017) identified a homozygous C-terminal deletion resulting in the complete loss of exon 8 and the partial loss of the carbohydrate-recognition domain [63] while Urquhart et al. (2016) reported a frame-shift mutation (deletion of two nucleotides), c.627_628delGC and p. (Ala213Leufs 5) [58]. Carriers of the above-mentioned mutations are suspected to be CL-K1-functionally-deficient. Two novel non-synonymous homozygous *COLEC11* mutations in 3MC patients were later described by Munye et al. (2017). One of them, c.309delT (p.Gly104Valfs29 in exon 4) again seems to predict premature appearance of a stop codon, while another one, c.G496A (p.Ala166Thr in exon 6), causes a change in the structure, affecting protein activity. Additionally, they found deletion of 10 nucleotides (89_98del ATGACGCCTG in exon 2) which predicted a frameshift change and the introduction of a premature stop codon (p.Asp30Alafs68) [7]. To summarize, 11 mutations have so far been described in the *COLEC11* gene in 17 3MC patients (Table 2). Six of them affect the CRD region of the collectin.

Three mutations of the *COLEC10* gene were found associated with 3MC syndrome: c.25 C>T (exon 1), c.226delA (exon 3), and c.528 C>G (exon 6). The last-mentioned results in severe impairment of protein secretion, whereas the two others lead to the nonsense-mediated decay of transcripts [7]. All mutations in the *COLEC10* gene occurring in patients with 3MC syndrome are shown in Table 3.

## 5. Concluding Remarks

The craniofacial disruptions observed in patients suffering from 3MC syndrome are similar to neural crest migration disorders. The proper migration of neural crest cells (NCC) is essential for the formation of bones, cartilage, ganglia, and muscles in the head [64]. Control and regulation of NCC migration is complex and involves many genetic pathways. Thus, there is a possibility that proteins with collagen-like regions linked to a CRD domain play dual roles, contributing to immunity and development. Certain complement components have previously been shown to play an essential role in cell migration. C3a and its C3aR receptor co-attracted crest cells, to coordinate migration in the first stages of NCC regulation [65]. Furthermore, surfactant protein D [66,67] and surfactant protein A [68] being collectins (although lacking complement activation property), have been also described as chemoattractants. Studies employing zebrafish [6] demonstrated that loss of CL-K1 and MASP-1/-3 is associated with craniofacial abnormalities. Both proteins were proposed to act as guidance cues for neural crest cell migration [47]. Moreover, CL-L1 was shown to regulate development of craniofacial structures acting as a migratory chemoattractant [7]. Three CL-K1 gene mutations associated with 3MC syndrome, resulting in Ser169Pro and Gly204Ser substitutions and Ser217 deletion, prevent normal secretion from mammalian cells due to structural changes caused by the failure to bind Ca2+ during biosynthesis. The destabilization of CRD probably leads to elimination of protein via the endoplasmic-reticulum-associated protein degradation pathway [47]. Gorelik et al. (2017) also reported that MASP-1 plays an important role in radial neuronal migration in the development of the cerebral cortex. Deficiency of MASP-1 and other components of the lectin pathway (C3 and MASP-2) leads to impairments in radial migration resulting in improper positioning of neurons and disorganized cortical layers [69]. Because CL-LK heterocomplexes were found to bind MASP-1 or MASP-3 homodimers via their collagen-like regions [47,70] it is possible that correct migration of neural crest cells requires cooperation between CL-LK and MASP-1/-3. This may be supported by the relationship between *MASP1/3*, *COLEC10,* and *COLEC11* mutations and the incidence of 3MC syndrome, associated with craniofacial abnormalities. During embryogenesis, these three genes are strongly expressed in the craniofacial cartilage, palatal structures, bronchi, heart, and kidneys and the corresponding proteins act as chemoattractants for the cranial crest nerve cells (crucial for the formation of the head skeleton), recognizing certain endogenous carbohydrate epitopes [6]. It should be however mentioned, that mice with knockout in *MASP1/3* or *COLEC11* genes developed normally and no defects similar to 3MC syndrome were noticed [71,72,73]. It moreover still remains to be clarified which defense pathways may compensate for MASP-1/3, CL-L1, and CL-K1 dysfunctions and why this rare disease is generally not accompanied by impaired immune response.

As mentioned, so far 11 mutations in the *COLEC11* gene, three in the *COLEC10* gene, and 16 in the *MASP1/3* gene associated with 3MC syndrome have been described. Further studies, however, are necessary to better understand the mechanisms by which dysfunction of *MASP1/3*, *COLEC10*, and *COLEC11* genes may lead to the 3MC syndrome.

## Figures and Tables

**Table 1:** Families affected by the Malpuech, Michels, Mingarelli, Carnevale (3MC) syndrome, associated with mutations in *MASP1/3* gene.

Family (Number of Carriers)	Origin	Nucleotide Change	Amino acid Change/Protein Change	References
F1 (2)	Turkey	c.2059G>A exon 12	p.Gly687Arg MASP-3 SP	Sirmaci et al., 2010 [5]
F2 (1)	Turkey	c.870G>A exon 6	p.Trp290* MASP-1/3 CUB1-EGF-CUB2-CCP1	
F3 (1)	Greece	c.1489 C>T exon 12	p.His497Tyr MASP-3 SP	Rooryck et al., 2011 [6]
F4 (2)	Italy	c.1888T>C exon 12	p.Cys630Arg MASP-3 SP	
F5 (1)	Brazil	c.1997G>A exon 12	p.Gly666Glu MASP-3 SP	
F6 (1)	Brazil	c.1997G>A exon 12	p.Gly666Glu MASP-3 SP	
F7 (1)	Turkey	c.1012-2A>G intron 7	Splice site mutation	Atik et al., 2015 [57]
F8 (1)	Turkey	c.891 +1G>T intron 6	Splice site mutation	
F9 (1)	Turkey	c.891 +1G>T intron 6	Splice site mutation	
F10 (1)	Pakistan	c.1451G>A exon 12	p. Gly484Glu MASP-3 SP	
F11 (1)	Turkey	c.1657G>A exon 12	p. Asp553Asn MASP-3 SP	
F12 (1)	Syria	c.1987G>T exon 12	p. Asp663Tyr MASP-3 SP	
F13 (1)	Israel	c.1987G>T exon 12	p.Asp663Tyr MASP-3 SP	Urquhart et al., 2016 [58]
F14 (1)	Sri Lanka	c.9G>A exon 2	p.Trp3* MASP-1/3 CUB1-EGF-CUB2-CCP1	
F14 (2)	Sri Lanka	c.760A>T exon 6	p.Leu 254* MASP-1/3 CUB1-EGF-CUB2-CCP1	
F15 (1)	India	c.547G>T exon 4	p.Val 183Leu MASP-1/3 CUB1-EGF-CUB2-CCP1	
F16 (1)	Germany	c.1993G>A exon 12	p.G665S MASP-3 SP	Pihl et al., 2017 [59]
F17 (1)	Pakistan	c.9G>A exon 2	p. Trp3* MASP-1/3 CUB1-EGF-CUB2-CCP1	Munye et al., 2017 [7]
F18 (1)	Turkey	c.1895_*1602+411del exon 12	p.Arg637Cysfs*1 MASP-3 SP	Graul-Neumann et al., 2018 [60]
F19 (3)	Turkey	c.2111T>G exon 12	p. Val704Gly MASP-1/MASP-3 CCP2	Basdemirci et al., 2019 [61]
F20 (1)	Syria	c.1987G>T exon 12	p.Asp663Tyr MASP-3 SP	Çakmaklı et al., 2019 [62]

**Table 2:** Families affected by 3MC syndrome, associated with mutations in the *COLEC11* gene.

Family (Number of Carriers)	Origin	Nucleotide Change	Amino Acid Change/Protein Change	References
F1 (2)	Tunisia	c.496T>C exon 8	p.Ser169Pro CRD	Rooryck et al., 2011 [6]
F2 (2)	Bangaldesh	c.45delC exon 2	p.Phe16SerfsX 85 N-terminal Collagen-like region	
F3 (2)	Afganistan	c.610G>A exon 8	p.Gly204Ser CRD	
F4 (1)	Saudi Arabia	c.648_650delCTC exon 8	p.Ser217del CRD	
F5 (1)	Pakistan	c.610G>A exon 8	p.Gly204Ser CRD	
F6 (1)	Italy	c.300delT exon 6	p.Gly101ValfsX 113 Neck domain	
F7 (1)	Italy	ex 1-3 deletion exons 1,2,3	Predicted: complete loss of N-terminus and partial loss of the collagen-like domains N-terminal collagen-like region	
F8 (2)	Israel	c.627_628delGC Exon 8	p. Ala213Leufs 5 CRD	Urquhart et al., 2016 [58]
F9 (2)	Pakistan	ex 8 deletion exon 8	Predicted: complete loss of C terminus and at least partial loss of the carbohydrate-recognition domain (CRD)	Gardner et al., 2017 [63]
F10 (1)	Pakistan	c.309delT exon 4	p.Gly104Valfs29 Collagen-like domain	Munye et al., 2017 [7]
F11 (1)	Somalia	c.G496A exon 6	p.Ala166Thr Neck domain	
F12 (1)	United Arab Emirates	89_98del ATGACGCCTG exon 2	p.Asp30Alafs68 N-terminal domain	

**Table 3:** Families affected by 3MC syndrome, associated with mutations in *COLEC10* gene.

Family (Number of Ccarriers)	Origin	Nucleotide Change	Amino Acid Change/Protein Change	References
F1 (2)	Pakistan	c.25C>T exon 1	p. Arg9Ter Signal peptide	Munye et al., 2017 [7]
		c.226delA exon 3	p.Gly77Glufs*66 Collagen-like region	
F2 (1)	Pakistan	c.25C>T exon 1	p. Arg9Ter Signal peptide	
		c.528C>G exon 6	p.Cys176Trp Neck domain	

## Abbreviations

3MC	Malpuech, Michels, Mingarelli, Carnevale syndrome
MASP	Mannose-binding lectin-associated serine proteases
MAp44	Mannose-binding lectin-associated protein, 44kDa
SP	Serine protease
BMP	Bone morphogenic protein
SNP	Single nucleotide polymorphism
MBL	Mannose-binding lectin
PRM	Pattern recognition molecule
TAFI	Thrombin-activatable fibrinolysis inhibitor
IL	Interleukin
MODS	Multiorgan dysfunction
SIRS	Systemic inflammatory response syndrome
LCOS	Low cardiac output syndrome
CRD	Carbohydrate-recognition domain
CL-L1	Collectin liver-1 (collectin-10)
CL-K1	Collectin kidney-1 (collectin-11)
SP-A	Surfactant protein A
SP-D	Surfactant protein D
LPS	Lipopolysaccharide
HIV	Human immunodeficiency virus
D-Man	D-MANNOSE
D-GlcNAc	N-ACETYL-D-GLUCOSAMINE
D-Gal	D-GALACTOSE
L-Fuc	L-FUCOSE
D-Fuc	D-FUCOSE
D-ManNAc	N-ACETYL-D-MANNOSAMINE
CRC	Colorectal cancer
MSCs	Mesenchymal stem cells

## References

[1] Mingarelli R., Scanderbeg A.C., Dallapiccola B. (1996). Two sisters with a syndrome of ocular, skeletal, and abdominal abnormalities (OSA syndrome). J. Med. Genet..

[2] Malpuech G., Deméocq F., Palcoux J.B., Vanlieferinghen P., Opitz J.M. (1983). A previously undescribed autosomal recessive multiple congenital anomalies/mental retardation (MCA/MR) syndrome with growth failure, lip/palate cleft(s), and urogenital anomalies. Am. J. Med. Genet..

[3] Michels V.V., Hittner H.M., Beaudet A.L. (1978). A clefting syndrome with ocular anterior chamber defect and lid anomalies. J. Pediatr..

[4] Carnevale F., Krajewska G., Fischetto R., Greco M.G., Bonvino A. (1989). Ptosis of eyelids, strabismus, diastasis recti, hip defect, cryptorchidism, and developmental delay in two sibs. Am. J. Med. Genet..

[5] Sirmaci A., Walsh T., Akay H., Spiliopoulos M., Şakalar Y.B., Hasanefendioğlu-Bayrak A., Duman D., Farooq A., King M.C., Tekin D. (2010). MASP1 Mutations in Patients with Facial, Umbilical, Coccygeal, and Auditory Findings of Carnevale, Malpuech, OSA, and Michels Syndromes. Am. J. Hum. Genet..

[6] Rooryck C., Díaz-Font A., Osborn D.P., Chabchoub E., Hernandez-Hernandez V., Shamseldin H., Kenny J., Waters A., Jenkins D., Al Kaissi A. (2011). Mutations in lectin complement pathway genes COLEC11 and MASP1 cause 3MC syndrome. Nat. Genet..

[7] Munye M.M., Diaz-Font A., Ocaka L., Henriksen M.L., Lees M., Brady A., Jenkins D., Morton J., Hansen S.W.K., Bacchelli C. (2017). COLEC10 is mutated in 3MC patients and regulates early craniofacial development. PLoS Genet..

[8] Degn S.E., Jensenius J.C., Thiel S. (2011). Disease-Causing Mutations in Genes of the Complement System. Am. J. Hum. Genet..

[9] Ammitzbøll C.G., Steffensen R., Nielsen H.J., Thiel S., Stengaard-Pedersen K., Bøgsted M., Jensenius J.C. (2013). Polymorphisms in the MASP1 Gene Are Associated with Serum Levels of MASP-1, MASP-3, and MAp44. PLoS ONE.

[10] Degn S.E., Hansen A.G., Steffensen R., Jacobsen C., Jensenius J.C., Thiel S. (2009). MAp44, a Human Protein Associated with Pattern Recognition Molecules of the Complement System and Regulating the Lectin Pathway of Complement Activation. J. Immunol..

[11] Fujita T. (2002). Evolution of the lectin—Complement pathway and its role in innate immunity. Nat. Rev. Immunol..

[12] Matsushita M., Endo Y., Fujita T. (2013). Structural and Functional Overview of the Lectin Complement Pathway: Its Molecular Basis and Physiological Implication. Arch. Immunol. Ther. Exp..

[13] Gregory L.A., Thielens N.M., Matsushita M., Sorensen R., Arlaud G.J., Fontecilla-Camps J.C., Gaboriaud C. (2004). The X-ray Structure of Human Mannan-binding Lectin-associated Protein 19 (MAp19) and Its Interaction Site with Mannan-binding Lectin and L-ficolin. J. Biol. Chem..

[14] Bork P., Beckmann G. (1993). The CUB Domain. A Widespread Module in Developmentally Regulated Proteins. J. Mol. Biol..

[15] Bally I., Rossi V., Thielens N.M., Gaboriaud C., Arlaud G.J. (2005). Functional Role of the Linker between the Complement Control Protein Modules of Complement Protease C1s. J. Immunol..

[16] Gaboriaud C., Gupta R.K., Martin L., Lacroix M., Serre L., Teillet F., Arlaud G.J., Rossi V., Thielens N.M. (2013). The Serine Protease Domain of MASP-3: Enzymatic Properties and Crystal Structure in Complex with Ecotin. PLoS ONE.

[17] Lynch N.J., Stover C.M., Sandrini S.M., Marston D., Presanis J.S., Schwaeble W.J. (2005). Composition of the Lectin Pathway of Complement in Gallus gallus: Absence of Mannan-Binding Lectin-Associated Serine Protease-1 in Birds. J. Immunol..

[18] Skjoedt M.-O., Hummelshoj T., Palarasah Y., Hein E., Munthe-Fog L., Koch C., Skjodt K., Garred P. (2011). Serum concentration and interaction properties of MBL/ficolin associated protein-1. Immunobiology.

[19] Troldborg A., Hansen A., Hansen S.W.K., Jensenius J.C., Stengaard-Pedersen K., Thiel S. (2017). Lectin complement pathway proteins in healthy individuals. Clin. Exp. Immunol..

[20] Dobó J., Pál G., Cervenák L., Gál P. (2016). The emerging roles of mannose-binding lectin-associated serine proteases (MASPs) in the lectin pathway of complement and beyond. Immunol. Rev..

[21] Krarup A., Gulla K.C., Gál P., Hajela K., Sim R. (2008). The action of MBL-associated serine protease 1 (MASP1) on factor XIII and fibrinogen. Biochim. Biophys. Acta BBA Proteins Proteom..

[22] Jenny L., Dobó J., Gal P., Schroeder V. (2015). MASP-1 of the complement system promotes clotting via prothrombin activation. Mol. Immunol..

[23] Krarup A., Wallis R., Presanis J.S., Gal P., Sim R. (2007). Simultaneous Activation of Complement and Coagulation by MBL-Associated Serine Protease 2. PLoS ONE.

[24] Gulla K.C., Gupta K., Krarup A., Gal P., Schwaeble W.J., Sim R., O’Connor C.D., Hajela K. (2009). Activation of mannan-binding lectin-associated serine proteases leads to generation of a fibrin clot. Immunology.

[25] Hess K., Ajjan R., Phoenix F., Dobó J., Gál P., Schroeder V. (2012). Effects of MASP-1 of the complement system on activation of coagulation factors and plasma clot formation. PLoS ONE.

[26] Dobó J., Major B., Kékesi K.A., Szabo I., Megyeri M., Hajela K., Juhász G., Závodszky P., Gál P. (2011). Cleavage of Kininogen and Subsequent Bradykinin Release by the Complement Component: Mannose-Binding Lectin-Associated Serine Protease (MASP)-1. PLoS ONE.

[27] Megyeri M., Jani P.K., Kajdácsi E., Dobó J., Schwaner E., Major B., Rigó J., Závodszky P., Thiel S., Cervenak L. (2014). Serum MASP-1 in complex with MBL activates endothelial cells. Mol. Immunol..

[28] Jani P.K., Schwaner E., Kajdácsi E., Debreczeni M., Ungai-Salánki R., Dobó J., Doleschall Z., Rigó J., Geiszt M., Szabó B. (2016). Complement MASP-1 enhances adhesion between endothelial cells and neutrophils by up-regulating E-selectin expression. Mol. Immunol..

[29] Jani P.K., Kajdácsi E., Megyeri M., Dobó J., Doleschall Z., Futosi K., Timár C.I., Mocsai A., Makó V., Gal P. (2014). MASP-1 Induces a Unique Cytokine Pattern in Endothelial Cells: A Novel Link between Complement System and Neutrophil Granulocytes. PLoS ONE.

[30] Cortesio C.L., Jiang W. (2006). Mannan-binding lectin-associated serine protease 3 cleaves synthetic peptides and insulin-like growth factor-binding protein 5. Arch. Biochem. Biophys..

[31] Skjoedt M.-O., Roversi P., Hummelshøj T., Palarasah Y., Rosbjerg A., Johnson S., Lea S.M., Garred P. (2012). Crystal Structure and Functional Characterization of the Complement Regulator Mannose-binding Lectin (MBL)/Ficolin-associated Protein-1 (MAP-1). J. Biol. Chem..

[32] Mortensen S.A., Skov L.L., Kjaer-Sorensen K., Hansen A.G., Hansen S.W.K., Dagnæs-Hansen F., Jensenius J.C., Oxvig C., Thiel S., Degn S.E. (2017). Endogenous Natural Complement Inhibitor Regulates Cardiac Development. J. Immunol..

[33] Mendes H.W., Boldt A.B.W., Stahlke E.V.R.S., Jensenius J.C., Thiel S., Messias-Reason I.J.T. (2020). Adding MASP1 to the lectin pathway—Leprosy association puzzle: Hints from gene polymorphisms and protein levels. PLoS Negl. Trop. Dis..

[34] Larsen J.B., Laursen M.A., Hvas C.L., Larsen K.M., Thiel S., Hvas A.-M. (2019). Reduced Mannose-Binding Lectin-Associated Serine Protease (MASP)-1 is Associated with Disturbed Coagulation in Septic Shock. Thromb. Haemost..

[35] Michalski M., Pągowska-Klimek I., Thiel S., Świerzko A.S., Hansen A.G., Jensenius J.C., Cedzynski M. (2019). Factors involved in initiation and regulation of complement lectin pathway influence postoperative outcome after pediatric cardiac surgery involving cardiopulmonary bypass. Sci. Rep..

[36] Fanelli G., Cordero A.G., Gardner P.J., Peng Q., Fernando M., Kloc M., Farrar C.A., Naeem A., Garred P., Ali R.R. (2017). Human stem cell-derived retinal epithelial cells activate complement via collectin 11 in response to stress. Sci. Rep..

[37] Henriksen M.L., Brandt J., Andrieu J.-P., Nielsen C., Jensen P.H., Holmskov U., Jørgensen T., Palarasah Y., Thielens N.M., Hansen S.W.K. (2013). Heteromeric Complexes of Native Collectin Kidney 1 and Collectin Liver 1 Are Found in the Circulation with MASPs and Activate the Complement System. J. Immunol..

[38] Hansen S., Selman L., Palaniyar N., Ziegler K., Brandt J., Kliem A., Jonasson M., Skjoedt M.-O., Nielsen O., Hartshorn K. (2010). Collectin 11 (CL-11, CL-K1) Is a MASP-1/3–Associated Plasma Collectin with Microbial-Binding Activity. J. Immunol..

[39] Selman L., Henriksen M., Brandt J., Palarasah Y., Waters A., Beales P., Holmskov U., Jørgensen T.J.D., Nielsen C., Skjodt K. (2012). An enzyme-linked immunosorbent assay (ELISA) for quantification of human collectin 11 (CL-11, CL-K1). J. Immunol. Methods.

[40] Bayarri-Olmos R., Hansen S.W.K., Henriksen M.L., Storm L., Thiel S., Garred P., Munthe-Fog L. (2015). Genetic Variation of COLEC10 and COLEC11 and Association with Serum Levels of Collectin Liver 1 (CL-L1) and Collectin Kidney 1 (CL-K1). PLoS ONE.

[41] Hansen S.W.K., Aagaard J.B., Bjerrum K.B., Hejbøl E.K., Nielsen O., Schrøder H.D., Skjoedt K., Sørensen A.L., Graversen J.H., Henriksen M.L. (2018). CL-L1 and CL-K1 Exhibit Widespread Tissue Distribution With High and Co-Localized Expression in Secretory Epithelia and Mucosa. Front. Immunol..

[42] Keshi H., Sakamoto T., Kawai T., Ohtani K., Katoh T., Jang S., Motomura W., Yoshizaki T., Fukuda M., Koyama S. (2006). Identification and characterization of a novel human collectin CL-K1. Microbiol. Immunol..

[43] Ohtani K., Suzuki Y., Eda S., Kawai T., Kase T., Yamazaki H., Shimada T., Keshi H., Sakai Y., Fukuoh A. (1999). Molecular Cloning of a Novel Human Collectin from Liver (CL-L1). J. Biol. Chem..

[44] Axelgaard E., Jensen L., Dyrlund T.F., Nielsen H.J., Enghild J.J., Thie L.S., Jensenius J.C. (2013). Investigations on collectin-liver 1 (CL-L1 or CL-10). J. Biol. Chem..

[45] Świerzko A.S., Michalski M., Sokołowska A., Nowicki M., Eppa Ł., Szala-Poździej A., Mitrus I., Szmigielska-Kapłon A., Sobczyk-Kruszelnicka M., Michalak K. (2018). The Role of Complement Activating Collectins and Associated Serine Proteases in Patients With Hematological Malignancies, Receiving High-Dose Chemotherapy, and Autologous Hematopoietic Stem Cell Transplantations (Auto-HSCT). Front. Immunol..

[46] Troegeler A., Lugo G., Hansen S.W.K., Rasolofo V., Henriksen M.L., Mori K., Ohtani K., Duval C., Mercier I., Bénard A. (2015). Collectin CL-LK Is a Novel Soluble Pattern Recognition Receptor for Mycobacterium tuberculosis. PLoS ONE.

[47] Girija U.V., Furze C.M., Gingras A.R., Yoshizaki T., Ohtani K., Marshall J.E., Wallis A.K., Schwaeble W.J., El-Mezgueldi M., Mitchell D.A. (2015). Molecular basis of sugar recognition by collectin-K1 and the effects of mutations associated with 3MC syndrome. BMC Biol..

[48] Hansen S.W.K. (2002). Lung Surfactant Protein D (SP-D) and the Molecular Diverted Descendants: Conglutinin, CL-43 and CL-46. Immunobiology.

[49] Henriksen M.L., Brandt J., Iyer S.S., Thielens N.M., Hansen S. (2013). Characterization of the interaction between collectin 11 (CL-11, CL-K1) and nucleic acids. Mol. Immunol..

[50] Selman L., Hansen S. (2012). Structure and function of collectin liver 1 (CL-L1) and collectin 11 (CL-11, CL-K1). Immunobiology.

[51] Smedbråten J., Sagedal S., Åsberg A., Hartmann A., Rollag H., Mjøen G., Fagerland M.W., Hansen S., Mollnes T.E., Thiel S. (2016). Collectin Liver 1 and Collectin Kidney 1 of the Lectin Complement Pathway Are Associated With Mortality After Kidney Transplantation. Am. J. Transplant..

[52] Troldborg A., Thiel S., Trendelenburg M., Friebus-Kardash J., Nehring J., Steffensen R., Hansen S., Laska M.J., Deleuran B., Jensenius J.C. (2018). The Lectin Pathway of Complement Activation in Patients with Systemic Lupus Erythematosus. J. Rheumatol..

[53] Storm L., Christensen I.J., Jensenius J.C., Nielsen H.J., Thiel S., The Danish Study Group on Early Detection of Colorectal Cancer (2014). Evaluation of complement proteins as screening markers for colorectal cancer. Cancer Immunol. Immunother..

[54] Farrar C.A., Tran D., Li K., Wu W., Peng Q., Schwaeble W., Zhou W., Sacks S.H. (2016). Collectin-11 detects stress-induced L-fucose pattern to trigger renal epithelial injury. J. Clin. Investig..

[55] Wu W., Liu C., Farrar C.A., Ma L., Dong X., Sacks S.H., Li K., Zhou W. (2017). Collectin-11 Promotes the Development of Renal Tubulointerstitial Fibrosis. J. Am. Soc. Nephrol..

[56] Nair A.R.K.G., Pandit H., Warty N., Madan T. (2014). Endometriotic mesenchymal stem cells exhibit a distinct immune phenotype. Int. Immunol..

[57] Atik T., Koparir A., Bademci G., Foster J., Altunoglu U., Mutlu G.Y., Bowdin S., Elcioglu N., Tayfun G.A., Atik S.S. (2015). Novel MASP1 mutations are associated with an expanded phenotype in 3MC1 syndrome. Orphanet J. Rare Dis..

[58] Urquhart J.E., Roberts R., De Silva D., Shalev S., Chervinsky E., Nampoothiri S., Sznajer Y., Revencu N., Gunasekera R., Suri M. (2016). Exploring the genetic basis of 3MC syndrome: Findings in 12 further families. Am. J. Med Genet. Part A.

[59] Pihl R., Jensen L., Hansen A.G., Thøgersen I.B., Andres S., Dagnæs-Hansen F., Oexle K., Enghild J.J., Thiel S. (2017). Analysis of Factor D Isoforms in Malpuech-Michels-Mingarelli-Carnevale Patients Highlights the Role of MASP-3 as a Maturase in the Alternative Pathway of Complement. J. Immunol..

[60] Graul-Neumann L.M., Mensah M.A., Klopocki E., Uebe S., Ekici A.B., Thiel C.T., Reis A., Zweier C. (2018). Biallelic intragenic deletion in MASP1 in an adult female with 3MC syndrome. Eur. J. Med. Genet..

[61] Basdemirci M., Sen A., Ceylaner S. (2019). Novel mutation in MASP1 gene in a new family with 3MC syndrome. Clin. Dysmorphol..

[62] Çakmaklı S., Kandur Y. (2019). 3MC syndrome: A case report. Arch. Clin. Exp. Med..

[63] Gardner O.K., Haynes K., Schweitzer D., Magee W.P., Urata M.M., Sanchez-Lara P.A., Johns A. (2017). Familial Recurrence of 3MC Syndrome in Consanguineous Families: A Clinical and Molecular Diagnostic Approach with review of the Literature. Cleft Palate Craniofacial J..

[64] Minoux M., Rijli F.M. (2010). Molecular mechanisms of cranial neural crest cell migration and patterning in craniofacial development. Development.

[65] Carmona-Fontaine C., Theveneau E., Tzekou A., Tada M., Woods M., Page K.M., Parsons M., Lambris J.D., Mayor R. (2011). Complement Fragment C3a Controls Mutual Cell Attraction during Collective Cell Migration. Dev. Cell.

[66] Cai G.-Z., Griffin G.L., Senior R.M., Longmore W.J., Moxley M.A. (1999). Recombinant SP-D carbohydrate recognition domain is a chemoattractant for human neutrophils. Am. J. Physiol. Content.

[67] Crouch E.C., Persson A., Griffin G.L., Chang D., Senior R.M. (1995). Interactions of pulmonary surfactant protein D (SP-D) with human blood leukocytes. Am. J. Respir. Cell Mol. Biol..

[68] Schagat T.L., Wofford J.A., Greene K.E., Wright J.R. (2003). Surfactant protein A differentially regulates peripheral and inflammatory neutrophil chemotaxis. Am. J. Physiol. Cell. Mol. Physiol..

[69] Gorelik A., Sapir T., Haffner-Krausz R., Olender T., Woodruff T.M., Reiner O. (2017). Developmental activities of the complement pathway in migrating neurons. Nat. Commun..

[70] Ma Y.J., Skjoedt M.O., Garred P. (2013). Collectin-11/MASP complex formation triggers activation of the lectin complement pathway—The fifth lectin pathway initiation complex. J. Innate Immun..

[71] Takahashi M., Iwaki D., Kanno K., Ishida Y., Xiong J., Matsushita M., Endo Y., Miura S., Ishii N., Sugamura K. (2008). Mannose-Binding Lectin (MBL)-Associated Serine Protease (MASP)-1 Contributes to Activation of the Lectin Complement Pathway. J. Immunol..

[72] Banda N.K., Takahashi M., Levitt B., Glogowska M., Nicholas J., Takahashi K., Stahl G.L., Fujita T., Arend W.P., Holers V.M. (2010). Essential Role of Complement Mannose-Binding Lectin-Associated Serine proteases-1/3 in the Murine Collagen Antibody-Induced Model of Inflammatory Arthritis. J. Immunol..

[73] Hwang I., Mori K., Ohtani K., Matsuda Y., Roy N., Kim Y., Suzuki Y., Wakamiya N. (2017). Collectin Kidney 1 Plays an Important Role in Innate Immunity against Streptococcus pneumoniae Infection. J. Innate Immun..

